# Pre-COVID-19-pandemic RSV epidemiology and clinical burden in pediatric primary care in Italy: a comparative analysis across two regions for the 2019/2020 season

**DOI:** 10.1186/s12879-024-09229-9

**Published:** 2024-04-11

**Authors:** Elisabetta Pandolfi, Daniela Loconsole, Maria Chironna, Jojanneke van Summeren, John Paget, Massimiliano Raponi, Luisa Russo, Ilaria Campagna, Ileana Croci, Carlo Concato, Carlo Federico Perno, Alberto Eugenio Tozzi, Giulia Linardos, Veronica Bartolucci, Sara Ciampini, Andrea Onetti Muda, Luigi De Angelis, Marta Luisa Ciofi Degli Atti, Caterina Rizzo

**Affiliations:** 1 Predictive and Preventive Medicine Research Unit, Bambino Gesù Children’s Hospital IRCCS, Rome, Italy; 2 Department of Interdisciplinary Medicine, University of Bari, Bari, Italy; 3 Nivel, Netherlands Institute for Health Services Research, Utrecht, The Netherlands; 4 Medical Direction, Bambino Gesù Children’s Hospital IRCCS, Rome, Italy; 5 Division of Metabolism, Bambino Gesù Children’s Hospital IRCCS, Rome, Italy; 6 Virology Unit, Laboratory Department, Bambino Gesù Children’s Hospital IRCCS, Rome, Italy; 7 Local Health Unit, Public Health Service, Rome, Italy; 8 Department of Laboratories, Bambino Gesù Children’s Hospital IRCCS, Rome, Italy; 9 Department of Translational Research and New Technologies in Medicine and Surgery, University of Pisa, Pisa, Italy; 10 Clinical Pathways and Epidemiology, Bambino Gesù Children’s Hospital IRCCS, Rome, Italy

**Keywords:** RSV, Infection surveillance, Acute respiratory infections, Primary care

## Abstract

**Background:**

Respiratory syncytial virus (RSV) infection in children under 5 years have a significant clinical burden, also in primary care settings. This study investigates the epidemiology and burden of RSV in Italian children during the 2019/20 pre-pandemic winter season.

**Methods:**

A prospective cohort study was conducted in two Italian regions. Children with Acute Respiratory Infection (ARI) visiting pediatricians were eligible. Nasopharyngeal swabs were collected and analyzed via multiplex PCR for RSV detection. A follow-up questionnaire after 14 days assessed disease burden, encompassing healthcare utilization and illness duration. Statistical analyses, including regression models, explored associations between variables such as RSV subtype and regional variations.

**Results:**

Of 293 children with ARI, 41% (119) tested positive for RSV. Median illness duration for RSV-positive cases was 7 days; 6% required hospitalization (median stay: 7 days). Medication was prescribed to 95% (110/116) of RSV cases, with 31% (34/116) receiving antibiotics. RSV subtype B and regional factors predicted increased healthcare utilization. Children with shortness of breath experienced a 36% longer illness duration.

**Conclusions:**

This study highlights a significant clinical burden and healthcare utilization associated with RSV in pre-pandemic Italian primary care settings. Identified predictors, including RSV subtype and symptomatology, indicate the need for targeted interventions and resource allocation strategies. RSV epidemiology can guide public health strategies for the implementation of preventive measures.

**Supplementary Information:**

The online version contains supplementary material available at 10.1186/s12879-024-09229-9.

## Introduction

Respiratory syncytial virus (RSV) is a common and highly contagious respiratory virus that affects all ages, but the highest clinical burden is in young children [1]. RSV is a leading cause of lower respiratory tract infections in children worldwide and it is the primary cause of bronchiolitis hospitalization in children under the age of five [2, 3]. RSV can also cause other serious complications, such as pneumonia, croup, and asthma exacerbations [4, 5]. In 2019, an estimated 6,6 million young children were infected by RSV, resulting in 1,4 million hospital admissions, 13,300 in-hospital deaths globally [6]. 

Infants under the age of one year are particularly susceptible to developing severe RSV infection, as their immune system is not fully developed [7]. Children with underlying medical conditions, such as premature birth, chronic lung disease, and congenital heart disease, are also at increased risk for severe RSV infection [8]. 

RSV infection is seasonal, with most cases occurring during the winter months in temperate climates [4]. Given that the epidemiology of RSV exhibits geographical and temporal variability, the burden associated with this respiratory infection may vary between regions and even within different populations within the same country [9, 10]. 

Primary care providers play a vital role in the diagnosis and management of RSV infection. They are often the first point of contact for families with children who are ill. Understanding the clinical burden of RSV infection in primary care settings can help to inform public health interventions and resource allocation [11–13]. 

Most European data on RSV epidemiology is gathered through existing influenza surveillance systems [11]. World Health Organization (WHO) and the European Center for Disease Prevention and Control (ECDC) advocate for an urgent need to develop and sustain population-based integrated surveillance systems for influenza, COVID-19, and other respiratory virus infections, including RSV [14]. Estimates of RSV burden usually rely on RSV-related hospitalization rates in children, and few studies have examined the impact of RSV infection in primary care settings. Information on the clinical burden of RSV infections, including primary care, is of utmost importance to comprehend the disease’s impact and to provide policymakers with appropriate information to introduce new monoclonal antibodies and vaccines [15]. Italy has implemented a robust national influenza surveillance system based on influenza-like illness case definition, but the collection of surveillance data on RSV has only recently started [16, 17].. Our study is built upon the protocol described by van Summeren et al. [17]. In their feasibility study they reported baseline characteristics of RSV patients in Italy and the Netherlands but did not dive into risk factors associated with severe RSV.

Given that the COVID-19 pandemic had a significant impact on the RSV epidemiological pattern of transmission [18], this study can provide contextual information to better understand the pre-pandemic epidemiology of RSV transmission in Italy and associated burden in primary care. Our findings can help to inform the development of preventive strategies, especially considering the new passive immunization strategies (monoclonal antibodies) and vaccines for RSV [19–21]. 

The objective of our study is to describe the epidemiology and clinical burden of RSV infection in children aged < 5 years in primary care settings in two Italian regions in the 2019/2020 season. We also sought to identify factors associated with severe RSV infection, defined as hospitalization or extra visits to healthcare facilities required.

## Materials and methods

### Study design

We conducted a multi-center prospective cohort study in primary care, as part of the RSV ComNet study, of which a detailed research protocol has been already published [17]. For each child (< 5 years) included in the study, data collection was performed on the day of swabbing (Day 0), and after approximately 14 days. The expected number of cases considered for this research was determined by referencing the WHO Strategy document, which recommends that countries strive to gather at least 500 respiratory samples annually from children under the age of 5 for effective RSV surveillance [22]. Since the RSV ComNet study served as a pilot for our research protocol, we opted to enroll a smaller number of children under the age of 5 at each location (approximately a total of 400).

### Study population

Patients were recruited during the winter season of 2019/20 (week 47-2019 to week 14-2020) via two networks of pediatricians working in primary care - one in Lazio Region (Central Italy) and the other in Apulia Region (Southern Italy). In Italy, the National Health System employs pediatricians to serve specific communities with certain sizes and demographics. The combined population of children < 5 in the two regions involved constituted 16.5% of the entire national population < 5.

Sentinel pediatricians recruited children with ARI symptoms, and after obtaining a signed informed consent from the parents to be included in the RSV ComNet Study, completed a questionnaire containing information on the patient demographics and clinical symptoms on the same day as the swab was taken. Children with a laboratory-confirmed diagnosis of RSV were followed up by telephone after 14 days (T14, supplementary online material).

### Case definition

Children < 5 years of age who consulted the pediatrician for symptoms of ARI were recruited in the study and underwent a nasopharyngeal swab. The ARI case definition was based on the WHO definition [23], and included the following criteria:

 [1] Acute– defined as a sudden onset of symptoms and [2] respiratory infection– defined as having at least one of the following: shortness of breath, cough, sore throat, coryza.

For the RSV ComNet study, we added the following inclusion criteria to the case definition: the symptoms had to be suggestive of infection according to the clinician’s judgment. Exclusion criteria were factors that could impair parent’s abilities to complete the follow-up telephone interviews, at least one of the following: insufficient Italian language proficiency, intellectual disabilities, and personal circumstances in the family, for example, a period of mourning.

### Laboratory procedures

The nasopharyngeal swabs were sent to a regional reference laboratory in Lazio and Apulia, together with the form completed by the pediatrician. Swabs were tested for 16 respiratory viruses (including RSV A and B, influenza virus A and B, human coronavirus OC43, 229E, NL-63 and HUK1, adenovirus, hRV, parainfluenza virus 1-2-3-4, human metapneumovirus-hMPV and human bocavirus-hBoV) through commercial multiplex RT-PCR kit (AllplexTM Respiratory Full Panel Assay, Seegene, South Korea).

Nucleic acids were extracted from a 200 μl sample of nasopharyngeal swabs and purified, using the EZ1 Virus Mini Kit v. 2.0 on the EZ1 Advanced XL platform (Qiagen, GmbH, Hilden, Germany). Nucleic acid extracts were eluted into 90 μl of buffer and processed immediately.

### Statistical analysis

Descriptive statistics were used to describe the clinical symptoms and healthcare use. Differences between age groups (1–12, 13–24 and 25–60 months) and regions (Lazio vs. Apulia) were analyzed using Wilcoxon Mann-Whitney, Kruskal-Wallis and Pearson’s Chi-square tests.

Healthcare use was defined as all extra visits to a healthcare facility after swab uptake. To investigate factors associated with high healthcare use, a uni- and multivariable logistic regression analysis was used to obtain odds ratios (ORs). For the duration of illness (measured in days) analysis, we carried out uni- and multivariable log-linear regression analysis, with a natural logarithmic transformation of the outcome variable due to its right-skewed distribution. For ease of interpretation, the coefficients obtained with the log-linear analysis were transformed according to the formula (eβ-1). When multiplied by 100, this value represents the percentage increase or decrease in the mean duration of illness for the index category compared to the reference. We defined a “high duration of illness” as an illness lasting more than 14 days. The factors that were assessed in the two models were based on what is known from the literature and data availability, and included: gender, age, being born in this year’s RSV season, prematurity, RSV subtype, clinical symptoms at baseline, and having a co-infection with another respiratory virus. In addition, the region was added to the model as healthcare is organized at a regional level in Italy. The univariable analyses were conducted for the two outcomes (healthcare useand duration of illness) and only the variables associated with at least one outcome in the univariable analyses were retained in the final models. All data analyses were carried out using STATA 14.1 SE (Stata Corp. College Station, Texas).

### Ethical considerations

The study was approved by the Medical Ethical Committee of Ospedale Pediatrico Bambino Gesù (OPBG) Medical Center (Prot. N 1301, 30th of September 2019).

## Results

**Fig. 1:**
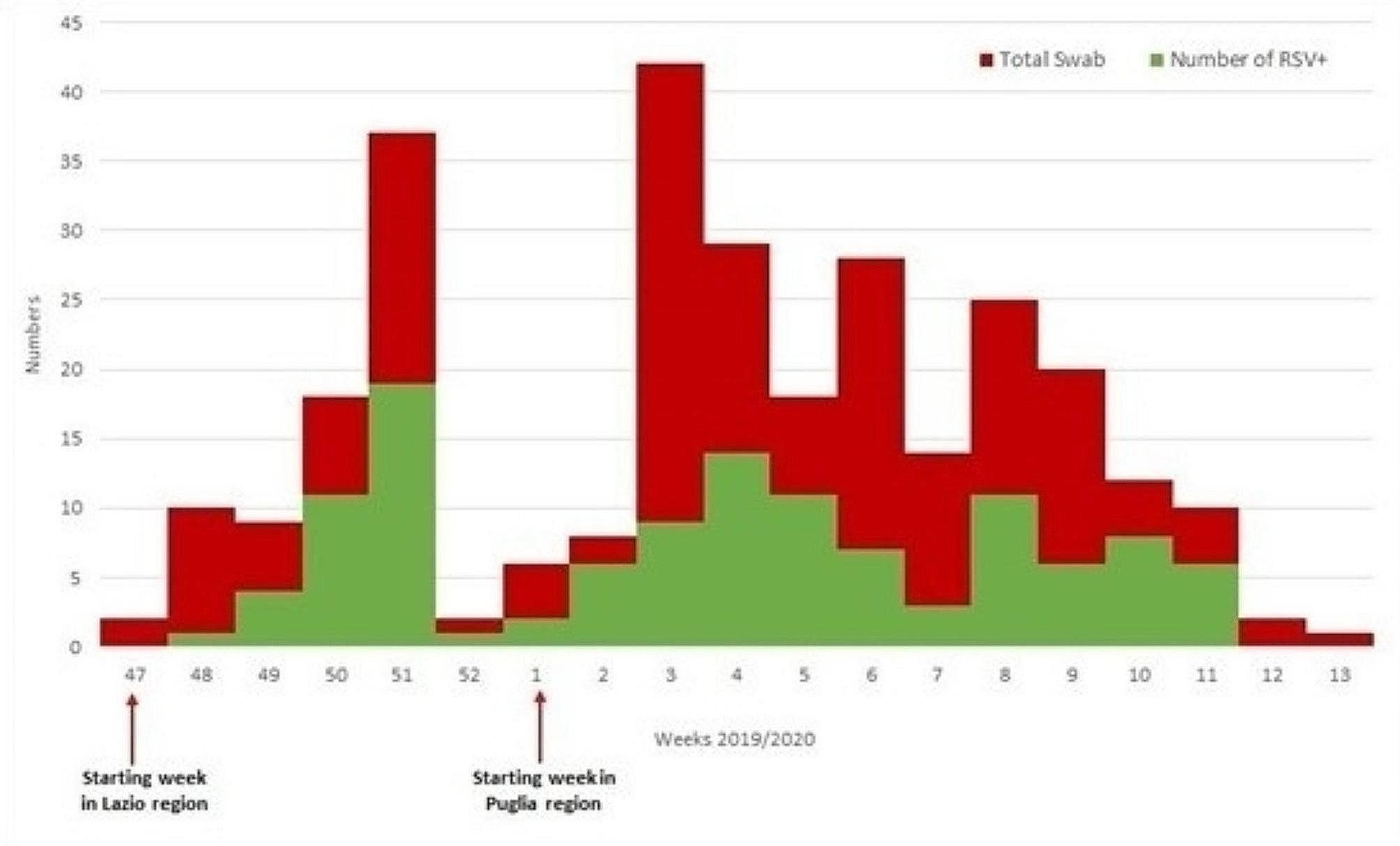
Distribution of number of swabs and positive RSV cases in Italy, week 47-2019 to 13-2020

Data collection started in week 47/2019 in the Lazio region and in week 01/2020 in the Apulia Region. Of the 15 sentinel pediatricians who were invited to participate 13 (86%) participated in the study. A total of 293 children with ARI symptoms were recruited for swabbing, and 119 (41%) tested RSV positive. The highest number of cases were recruited in week 51/2019 and 4/2020 (Fig. 1). In total 168 children (57%) were recruited in Lazio, and 125 (43%) in Apulia. One hundred and thirty patients (44%) were younger than 1 year of age, 70 (24%) were in the 13–24 months age group and 93 (32%) were in the 25–60 months age group. Children resident in Apulia region were more likely to be RSV positive compared to children in the Lazio region (*p* < 0.001). All the 119 RSV cases were followed up for 14 days.

### Clinical characteristics of RSV cases

Baseline characteristics of RSV-positive children are shown in Table 1. 50% of the children were boys and the median age was 15 months (IQR range: 7–29). The proportion of RSV-A subtype was 76%, with a higher proportion of RSV-A isolated in Lazio region compared to Apulia (87% vs. 67%). The proportion of RSV cases testing positive for another respiratory virus was 51%, this proportion was not statistically significantly different between regions or age groups. Among the identified respiratory viruses, the most frequent were Rhinovirus (*n* = 42, 35%), Adenovirus (*n* = 10, 8%), Enterovirus (*n* = 8, 7%), OC43 (*n* = 7, 6%), Bocavirus (*n* = 5, 4%) and Influenza A/H3N2 virus (*n* = 2, 2%).

The most frequently reported symptoms at inclusion were cough (98%), coryza (89%), shortness of breath (76%), and sore throat (30%). After 14 days, 66% (*n* = 76) of the parents reported their child did not have any remaining symptoms and 92% returned to normal daily activities. The most reported remaining symptoms were dry cough (17%), nose complaints (17%), and wheezing (10%). The median duration of illness was 7 days (IQR 5–10) and is not significantly different among age groups and regions.

Among symptoms at inclusion, only shortness of breath differed by age, with 87% of children under one year of age reporting this symptom, a prevalence of 73% between children over 2 years, and only 60% of children with an age between one and two years. (*p*-value = 0.031). Symptoms at initial assessment did not differ by region. Results are reported in supplementary table S1.

At least one persisting symptom at Day 14 was present in 34% of RSV-positive children. These symptoms did not differ by age, and only wheezing differed by region ( 10 in Lazio (19%) and 2 in Apulia (3%), *p*-value = 0.015). Results are reported in supplementary material table S2.

**Table 1:** Baseline characteristics of RSV positive children included in RSV ComNet

		Age categories				Region		
	Total (*n* = 119)	1–12 months (*n* = 53)	13–24 months (*n* = 26)	25–60 months (*n* = 40)	*p*	Lazio (*n* = 55)	Apulia (*n* = 64)	*p*
Boy (n, %)	59 (50%)	30 (57%)	13 (50%)	16 (40%)	0.284	25 (45%)	34 (53%)	0.404
Age in months (median IQR)	15 (–29)	6 (4–9)	19.5 (15–21)	35 ( 30–46)	-	14 (–32)	15 (6–29)	0.244
Prematurity (n, %)	6 (5%)	0 (-)	4 (15%)	2 (5%)	**0.014**	2 (4%)	4 (6%)	0.530
Co-infection with at least one virus* (n, %)	61 (51%)	26 (49%)	14 (54%)	21 (53%)	0.906	31 (56%)	30 (47%)	0.302
RSV B (n, %)	28 (24%)	13 (25%)	5 (19%)	10 (25%)	0.842	7 (13%)	21 (33%)	**0.010**

*Samples were tested for 16 co-viruses. None of the children had a history of malnutrition, immunocompromised, or previous RSV infection in this season. One child (1%) had another chronic respiratory disease, and 2 children (2%) had another chronic medical condition

### Healthcare use

The healthcare usage of children with an RSV infection is shown in Table 2. The proportion of children who consulted the Emergency Department was significantly higher in the Apulia region (23%) compared to Lazio (6%). Children in the Apulia region consulted and contacted their pediatrician significantly more often compared to the children in Lazio. Overall, 110 children (95%) had medication prescribed, which was frequently an antibiotic (31%, 34/110). Although the proportion of children that got a medication prescribed was comparable between regions, there are some significant differences in the type of medication prescribed. In the Apulia region the use of paracetamol (52%) was significantly higher compared to Lazio (31%). In contrast, in the Lazio region, the use of other medication (i.e. bronchodilators, aerosol therapy, nasal washes, syrups for cough, steroids, etc.) was significantly higher (98% vs. 75%). Although not statistically significant, children < 1 year old were more often referred to the hospital (e.g. access to the emergency department or hospitalized after consultation) compared to older children. Also, children > 1 year old more often received pain medications and antibiotics compared to children < 1 year of age. While children < 1 year of age received other medications (including all types of cough syrup) compared to the older age groups. None of the enrolled children was hospitalized 14 days after the swab was taken.

**Table 2:** Healthcare use of RSV infections in young children in the 14 days after enrollment

	Total (*n* = 116)	1–12 months (*n* = 52)	13–24 months (*n* = 25)	25–60 months (*n* = 39)	p*	Lazio (*n* = 52)	Apulia (*n* = 64)	p*
Healthcare usage^$^	87 (75%)	45 (87%)	18 (72%)	24 (62%)	**0.024**	31 (60%)	56 (88%)	**0.001**
Number of consultation to pediatrician after swab *(median, IQR)*	1 (0–2)	2 (1–3)	1(0–2)	1 (0–2)	0.094	1 (0–2)	2 (–3)	**< 0.001**
Number ofphone call/email after swab	1 (0–2)	1 (1–3)	1 (0–2)	1 (0–2)	0.861	1 (0–2)	(1–3)	**0.025**
Consultation with another doctor (sub-specialist) after swab *n (%)*	11 (9%)	5 (10%)	1 (4%)	5 (13%)	0.501	5 (10%)	6 (9%)	0.965
Emergency department *n (%)*	18(16%)	11 (21%)	3 (12%)	4 (10%)	0.314	3 (6%)	15 (23%)	**0.009**
Hospitalizations *n (%)*	7 (6%)	6 (12%)	0 (-)	1 (3%)	0.074	2 (4%)	5 (8%)	0.372
Days of hospitalizations *(median, IQR)*	7 (3–9)	7 (3–9)	-	4 (4–4)	0.611	2.5 (–3)	7 (7–9)	**0.049**
**Use of medications and prophylaxes**								
Paracetamol	47 (43%)	14 (30%)	16 (64%)	17 (44%)	**0.024**	15 (31%)	32 (52%)	**0.021**
Other pain medication (e.g. ibuprofen)	4 (4%)	0 (-)	0 (-)	4 (10%)	**0.023**	2 (4%)	2 (3%)	0.823
Antibiotics	34 (31%)	8 (17%)	10 (40%)	16 (41%)	**0.034**	15 (31%)	19 (31%)	0.952
Bronchodilators	38 (31.9%)	18 (34%)	10 (38.6%)	10 (25%)	0.160	38 (69.1%)	0	< 0.001
Steroids	6 (5%)	2 (3.8%)	3 (11.5%)	1 (2.5%)	**0.120**	6 (10.9%)	0	**0.009**
Cough syrup	4 (3.4%)	0	4 (15.4%)	0	**0.001**	4 (7.3%)	0	**0.034**
Use of any medication^	110 (95%)	46 (88%)	25 (100%)	39 (100%)	**0.020**	49 (94%)	61 (95%)	0.794
Palivizumab *n (%)*	0 (-)	0 (-)	0 (-)	0 (-)	-	0 (-)	0 (-)	-

*p < 0.05 $ Healthcare usage was defined as at least one of the following variables: consultation to PCP after swab, home visits by PCP, consultation with another doctor after swab, access to emergency department and hospitalizations. ^ Medication included antibiotics, paracetamol, other pain medication (e.g. ibuprofen) and other medication. None of the patients reported home visits, intensive care unit admission, or paramedical care at day 14. These outcomes were not reported in the table.

### Predictors of healthcare usage and duration of illness

The predictors of healthcare usage and duration of illness based on the log-linear and logistic regression analysis are shown in Tables 3 and 4. For the duration of illness, only shortness of breath was a significant predictor (*p* = 0.023, 95% CI 0.041–0.786): children presenting with shortness of breath had a 36% increase in the duration of illness compared to those not having shortness of breath (Table 3). For high healthcare use, the following predictors were significant in the multivariate model: age, region, and RSV subtype B. Children from the Apulia region had 5-fold higher odds of using healthcare facilities compared to children from the Lazio region (OR = 5.0; *p* = 0.003, 95% CI 1.7–14.9) (Table 4). 92% of the children returned to their normal daily activities after 14 days.

**Table 3:** Predictors for high duration of illness (log-linear regression analysis, *n* = 115)

	Duration of illness*					
	Univariable analysis			Multivariable analysis		
	e^β^-1	(95%CI)	*p*	e^β^-1	(95%CI)	*p*
Age in months	0.003	-0.095-0.051	0.473	-0.008	-0.098-0.092	0.873
Region (Puglia)	-0.086	-0.274-0.150	0.454	-0.166	-0.344-0.059	0.136
Shortness of breath	**0.363**	0.041–0.786	**0.023**	**0.382**	0.048–0.823	**0.022**
RSV subtype B	0.234	-0.058-0.616	0.130	0.298	-0.020-0.718	0.068

*Variables associated with at least one outcome (duration of illness of healthcare use) were retained in the final models

**Table 4:** Predictors for healthcare usage(*n* = 116)

	Healthcare use*					
	Univariable analysis			Multivariable analysis		
	OR	(95%CI)	*p*	OR	(95%CI)	*p*
Age in months	**0.96**	0.9-1.0	**0.002**	**0.54**	0.36–0.8	**0.002**
Region (Puglia)	**4.7**	1.9–12.0	**0.001**	**5.4**	2.0-14.9	**0.001**
Shortness of breath	1.8	0.7–4.6	0.229	0.99	0.33-3.0	0.986
RSV subtype B	**5.4**	1.2–24.6	**0.028**	3.7	0.8–16.1	0.082

*Variables associated with at least one outcome (duration of illness or healthcare use) were retained in the final models.

## Discussion

Our study describes the clinical burden and healthcare use in young children aged < 5 years with RSV in the community at large through the analysis of consultations with primary care pediatricians in Italy. Our findings highlighted that in primary care half of the children with RSV had a duration of illness of at least seven days and 75% of children had at least one additional consultation with their pediatrician, another medical specialist, or at the emergency department. The median age of 15 months in our sample, underlines different epidemiology of RSV in primary care compared to the hospital setting, where the median age of RSV cases is considerablylower. Children under 12 months are more often referred to the hospital compared to children aged > 12 months, this might explain why no prematurely born children under one year of age were recruited in this study in the primary care setting. Children older than 12 months receive pain medications and antibiotic prescriptions more often than children aged < 12 months; on the other hand, the number of prescriptions of other general medications (e.g., cough syrup) was higher in children < 12 months, but these differences were small.

The healthcare usage of RSV-infected children in primary care is lower than that reported in hospital settings; however, the number of children with an RSV infection in the primary care setting is much higher.

Healthcare use is only one aspect of the burden of disease, which is a broad concept encompassing both clinical and socio-economic factors. Infact, the outpatient burden of RSV on healthcare resources is not fully recognized by healthcare providers and policymakers, there is a need for more studies to measure not only the clinical burden and healthcare utilization, but also thesocio-economical impact of RSV infections in young children in primary care. More detailed estimates of RSV-associated burden in primary care are necessary to provide a benchmark to evaluate the benefits associated with new immunization strategies or treatments [2, 15, 24]. In Italy, the National Health System is a federal system in which 20 Italian regions have the responsibility for the organization and administration of publicly financed healthcare systems [25, 26]. This might explain the differences in healthcare and medication use between the Lazio and Apulia regions. This agrees with other studies reporting differences in healthcare utilization across the country, depending on several factors, among which, the different availability of doctors is also a prerequisite for the use of treatments [27]. The differences between regional healthcare systems in Italy, also in primary care organizations, are complex and require specific investigations, which are beyond the scope of our paper. We showed that a significant number of RSV outpatient visits occur among primary care practices with 119 RSV-positive children out of 293 children with ARI (41%); the illness reflects moderate to severe disease with symptoms like shortness of breath (76%) and wheezing (10%). This suggests that the severity of disease in primary care settings may be underestimated and that outpatient visits contribute to RSV’s burden in terms of clinical symptoms and healthcare usage [1, 13]. 

The temporal pattern of RSV activity is different across regions of Italy, with RSV activity in the south of the country appearing later and persisting for a longer period (until April– June). In addition, the number of hospital admissions has been observed to be over four times higher in the south compared to central or northern Italy. It’s worth noticing that the highest probability of finding an RSV-positive swab in the Apulia region might be explained by the later start of recruitment in that region.

The significant impact of RSV on young children underscores the need for ongoing efforts to create new interventions for RSV, such as vaccines, antiviral monoclonal antibodies (mAbs), and treatments. One currently available preventive measure against RSV infection is palivizumab, an injectable monoclonal antibody. While it is considered cost-effective for specific high-risk infant groups, it necessitates monthly vaccinations during the winter months, and treatment options are generally limited to supportive care. [28, 29, 30, 31] There have been several promising RSV vaccine candidates and mAbs with extended half-life times in advanced clinical trials for some time now [32, 33]. Notably, a bivalent recombinant RSV vaccine was recently approved by the EMA for passive immunization of infants from birth through 6 months of age following its administration to the mother during pregnancy. Children under the age of 2 are a key target group for mAbs or vaccination, given their high risk of severe RSV-related illness and their role in the transmission of RSV within the community. [33] This is one of the first studies that prospectively measures the clinical burden of RSV infection and healthcare usage of children infected with RSV, aged less than 5 years in primary care. In addition, to our knowledge, only a few studies have measured the clinical burden of RSV infections in primary care [34, 35, 36]. One limitation of the study is that we measured the clinical burden and healthcare usage only over 14 days. Future studies may consider measuring the burden over a longer period and include complications associated with the RSV infection, like otitis media or pneumonia, or long-term consequences like asthma or wheezing. The duration of illness was self-reported, which makes it prone to recall bias. However, 92% of the children returned to their normal daily activities after 14 days.

Moreover, we acknowledge that RSV infection can cause mild symptoms that do not require medical attention and that more severe RSV infection might not be detected in surveillance focusing only on the primary care setting. There was an important reduction in enrollments during the 2 weeks over the Christmas holidays in which we may have lost cases, this could be explained by the fact that pediatricians were less likely to enroll patients who could be difficult to follow-up in the subsequent 14 days period or by a reduction of ARI cases due to fewer social interaction related to school holidays.

There was a premature stop to the study, therefore children were not tested for RSV after March 2020; therefore COVID-19 pandemic may have influenced the healthcare utilization of the children because children who were enrolled late into the study were not able to seek healthcare or maintain their normal healthcare utilization practices because of the SARS-CoV-2 pandemic. This unexpected event contributed to making the study underpowered, as the objective of 400 enrolled children was not met. This limitation could affect the robustness of our findings, in particular when we observed an absence of correlation. It was difficult to compare the clinical symptoms of children measured at enrollment and after 14 days because the measured symptoms differed slightly, (i.e., shortness of breath versus wheezing). We recommend measuring the same symptoms at both time points for future studies. Unfortunately, for the 2019/2020 season, we do not have additional data on the RSV circulation in primary care in Italy. Therefore, we are unable to provide information about the exact timing of the RSV peak.

Another potential limitation of this study is that we excluded children whose parents or caregivers had insufficient proficiency in the Italian language. This exclusion could result in an underrepresentation of various ethnicities, especially given that we did not assess this factor in the questionnaire.

## Conclusions

This study explores the clinical burden and healthcare utilization among young children with RSV infection in primary care settings. A notable number of RSV-related outpatient visits took place in primary care practices, with half of these cases exhibiting an illness duration of at least seven days, and 6% (rising to 12% among infants under 12 months) required hospitalization. Overall, in the Apulia region, RSV cases were more severe, with infected children having a higher probability of using healthcare facilities compared to the Lazio region. The elucidation of RSV epidemiology before the onset of the COVID-19 pandemic holds significant importance as a baseline for future studies that aim to examine any changes that may have occurred in the post-pandemic era.

### Electronic supplementary material

Below is the link to the electronic supplementary material.

Supplementary Material 1

## Data Availability

The datasets generated and/or analyzed during the current study are not publicly available due to ethical and privacy restrictions but are available from the corresponding author on reasonable request.
