# Supplementary material for: Pre-COVID-19-pandemic RSV epidemiology and clinical burden in pediatric primary care in Italy: a comparative analysis across two regions for the 2019/2020 season
Source: BMC Infect Dis. 2024 Apr 11;24:388. doi: 10.1186/s12879-024-09229-9 (PMC11007985; doi:10.1186/s12879-024-09229-9)
Supplement: Supplementary file 1 — Supplementary Material 1 [file 12879_2024_9229_MOESM1_ESM.docx]

**SUPPLEMENTARY**
**Table S1.** Baseline symptoms of children with a RSV infections.

|  | **Baseline symptoms (Day 1)*** | | | |  |
| --- | --- | --- | --- | --- | --- |
|  | Shortness  of breath (n,%) | Cough  (n,%) | Sore throat  (n,%) | Coryza  (n,%) | At least one persisting symptom  (n,%) |
| Total (n=119) | 89 (76%) | 117 (98%) | 36 (30%) | 106 (89%) | 40 (34%) |
| **Age categories** |  |  |  |  |  |
| 1-12 months (n=53) | 45 (87%) | 52 (98%) | 16 (30%) | 46 (87%) | 21 (41%) |
| 13-24 months (n = 26) | 15 (60%) | 26 (100%) | 10 (38%) | 26 (100%) | 8 (32%) |
| 25-60 months (n = 40) | 29 (73%) | 39 (98%) | 10 (25%) | 34 (85%) | 11 (28%) |
| p-value* | **0.031** | 0.733 | 0.508 | 0.125 | 0.417 |
| **Region** |  |  |  |  |  |
| Lazio (n=55) | 38 (72%) | 53 (96%) | 20 (36%) | 50 (91%) | 20 (38%) |
| Apulia (n=64) | 51 (80%) | 64 (100%) | 16 (25%) | 56 (88%) | 20 (32%) |
| p-value* | 0.313 | 0.124 | 0.178 | 0.552 | 0.452 |

* p<0.05. Note: We did not collect information about fever as fever is not included in the WHO ARI case definition.

**Table S2.** Persistent symptoms of children with RSV infections.

|  |  | **Persistent symptoms (Day 14) **** | | | |  |  |
| --- | --- | --- | --- | --- | --- | --- | --- |
|  | **At least one persisting symptom**  **(n,%)** | **Wheezing/**  **whistling in the chest**  **(n,%)** | **Persistent cough with slime**  **(n,%)** | **Persistent dry cough**  **(n,%)** | **Nose complaints**  **(n,%)** | **Duration of illness (days)**  **(median and IQR)** | **Return to normal daily activities** |
| Total (n=119) | 40 (34%) | 12 (10%) | 6 (5%) | 20 (17%) | 20 (17%) | 7 (5-10) | 106 (92%) |
| **Age categories** |  |  |  |  |  |  |  |
| 1-12 months (n=53) | 21 (41%) | 7 (13%) | 4 (8%) | 10 (19%) | 10 (19%) | 8 (7-10) | 49 (96%) |
| 13-24 months (n = 26) | 8 (32%) | 2 (8%) | 2 (8%) | 2 (8%) | 7 (28%) | 7 (5-10) | 22 (88%) |
| 25-60 months (n = 40) | 11 (28%) | 3 (8%) | 0 (-) | 8 (21%) | 3 (8%) | 7 (5-10) | 35 (90%) |
| p-value* | 0.417 | 0.772 | 0.401 | 0.256 | 0.106 | 0.648 | 0.368 |
| **Region** |  |  |  |  |  |  |  |
| Lazio (n=55) | 20 (38%) | 10 (19%) | 4 (8%) | 11 (21%) | 13 (25%) | 7 (5-13) | 47 (90%) |
| Apulia (n=64) | 20 (32%) | 2 (3%) | 2 (3%) | 9 (14%) | 7 (11%) | 7 (5-10) | 59 (94%) |
| p-value* | 0.452 | **0.015** | 0.848 | 0.587 | 0.117 | 0.573 | 0.516 |

* P<0.05. **3 children did not complete the Day-14 follow-up questionnaire, one child in each age category.
